# MicroRNA-203 inhibits cell proliferation by repressing ΔNp63 expression in human esophageal squamous cell carcinoma

**DOI:** 10.1186/1471-2407-11-57

**Published:** 2011-02-07

**Authors:** Yang Yuan, Zhi-Yong Zeng, Xiao-Hong Liu, De-Jun Gong, Jing Tao, He-Zhong Cheng, Sheng-Dong Huang

**Affiliations:** 1 Institute of Cardiothoracic Surgery, Changhai Hospital, Second Military Medical University, Shanghai, PR China; 2 Department of Cardiothoracic Surgery, Fuzhou General Hospital of Nanjing Command, PLA. Fujian, PR China; 3 Department of Cardiothoracic Surgery, Changhai Hospital, Second Military Medical University, Shanghai, PR China

## Abstract

**Background:**

This study was performed to investigate the effect of microRNA-203 (miR-203) and ΔNp63 on cell proliferation and the functional connection between miR-203 and ΔNp63 in ESCC.

**Methods:**

We employed 2 human ESCC cell lines, Eca109 and TE-1, as the model system. The effect of miR-203 and ΔNp63 on cell proliferation was determined in cells transfected with miR-203 mimic and ΔNp63 small interfering RNA (siRNA), respectively. The regulation of ΔNp63 expression in ESCC cells by miR-203 was studied by luciferase reporter assay, RT-PCR and western blot analysis in cells transfected with miR-203. The effect of ΔNp63 re-expression on miR-203 induced inhibition of cell proliferation was studied by cell proliferation assay in cells cotransfected with miR-203 and pcDNA-ΔNp63 plasmid (without the 3'-UTR of *ΔNp63*).

**Results:**

We found that both miR-203 and ΔNp63 siRNA signicantly inhibited cell proliferation in ESCC. MiR-203 could down-regulate endogenous ΔNp63 expression at the posttranscriptional level. Moreover, re-expression of ΔNp63 in cells transfected with miR-203 significantly attenuated the miR-203 induced inhibition of cell proliferation.

**Conclusions:**

Our data implied that miR-203 could inhibit cell proliferation in human ESCC through ΔNp63-mediated signal pathway. Therefore, we propose that miR-203 might be used as a therapeutic agent for human ESCC.

## Background

Esophageal cancer is one of the most lethal human cancers mainly located in China, Japan, and southeast Africa [1]. According to the etiologic and pathologic characteristics, it could be divided into two main forms, esophageal squamous cell carcinoma (ESCC) and esophageal adenocarcinoma (EAC). Previous reports showed that the incidence of ESCC is much higher than that of EAC in the above mentioned areas [1,2], and the 5-year survival rate of ESCC after surgery ranges from 14% - 22% [3,4]. Much effort has been spent on the study of the biological behavior of ESCC cells to develop effective treatment strategies. Although some oncogenes and tumor suppressor genes were reported to be associated with the development of ESCC [5-7], few specific molecules regulating the initiation and progression of ESCC have been identified. Conceivably, elucidation of the molecular pathways involved in the cell proliferation of ESCC will provide important clues for the development and evaluation of novel anticancer therapies.

MicroRNAs, a class of small non-coding RNAs, have been identified as a new kind of gene expression regulators through targeting the 3'-untranslated region (UTR) of mRNAs for translational repression, degradation or both [8-10]. In the recent years, mounting data suggest that microRNAs are involved in essential tumor cell biological processes, such as proliferation, invasion, and apoptosis [9,11,12].

It was reported that MicroRNA-203 (miR-203) located in a region at chromosome 14 [13], which contains a high density of microRNAs (including about 12% of the known human microRNA gene), exhibited significantly down-regulated expression in some tumors such as head and neck squamous cell carcinomas [14], hematopoietic malignancy [13] and colon cancer [15]. Subsequent studies showed that the expression level of miR-203 is inversely correlated with the capacity of cell proliferation in human head and neck squamous cell carcinoma [14], hepatocellular carcinoma [16], chronic myelogenous leukemia and B cell leukemia [13]. In addition, ΔNp63, an important oncogene regulating cell proliferation in some tumors [17], was recently identified as a target gene of miR-203 in human epithelial precursor cells, as well as human head and neck squamous cell carcinoma cells [14,18]. These findings suggested that there might be a functional connection between miR-203 and ΔNp63 in cell proliferation regulation.

In human ESCC, genome-wide microRNA expression profile assay showed that the expression level of miR-203 was significantly down-regulated in tumor tissue compared with the matched normal tissue [19-21]. In contrast, the expression level of ΔNp63 was significantly higher in tumor tissue than in the matched normal tissue [22,23]. However, the effect of miR-203 and ΔNp63 on the proliferation of ESCC cells, as well as the functional relationship between miR-203 and ΔNp63 in ESCC cells has not been documented. Since certain microRNAs may show different functions in different type of tumors [24], the role of miR-203 in the regulation of cell proliferation of ESCC warrants investigation.

In light of the previous reports, we hypothesized that miR-203 might regulate the proliferation of ESCC cells through the ΔNp63-mediated signal pathway. Therefore, using 2 human ESCC cell lines (Eca109 and TE-1) as a model system, here we set out to investigate the effect of miR-203 and ΔNp63 on the proliferation of ESCC cell, as well as the regulation of the expression of ΔNp63 by miR-203 in ESCC. Our results suggest that miR-203 may inhibit the cell proliferation in ESCC through the ΔNp63-mediated signal pathway.

## Methods

### Cell culture

Human ESCC cell lines Eca109 and TE-1 were purchased from the Shanghai Institute of Biochemistry and Cell Biology (Shanghai, China). Cells were maintained in RPMI1640 (Invitrogen) supplemented with 10% fetal bovine serum (Invitrogen), 100 U/ml penicillin and 100 μg/ml streptomycin, within a humidified atmosphere containing 5% CO_2 _at 37°C.

### Cell transfection

1 × 10^6 ^cells cultured in a well of 6-well cell culture plate were transiently transfected with 50 pmol of miR-203 double-stranded mimics (or control microRNA) and ΔNp63 siRNA oligonucleotide duplexes (or control siRNA) using Lipofectamine 2000 (Invitrogen) according to the manufacturer's protocol, respectively. Transfection efficiency was optimized using 6-carboxyfluorescein-labeled microRNA (or siRNA) at approximately 80% in Eca109 and TE-1 cells.

The sequences of miR-203 were:

Sense: 5'-GUGAAAUGUUUAGGACCACUAG-3',

Anti-sense: 5'-CUAGUGGUCCUAAACAUUUCAC-3',

A scrambled microRNA with no homology to any known human microRNA was used as negative control:

Sense: 5'-GUUGAACUGUUAAGAACCACUGG-3',

Anti-sense: 5'-CCAGUGGUUCUUAACAGUUCAAC-3',

The siRNA oligonucleotides targeting ΔNp63 were designed as previously described [25]:

Sense: 5'-AACAGCCAUGCCCAGUAUGUA-3';

Anti-sense: 5'- UACAUACUGGGCAUGGCUGUU-3'.

A scrambled siRNA with no homology to any known human mRNA was used as negative control:

Sense: 5'-CCCUGUUAAAAAUCCAGGCGA-3';

Anti-sense: 5'-UCGCCUGGAUUUUUAACAGGG-3'.

All microRNA mimics or siRNA oligonucleotide duplexes were synthesized by Genephama Biotech (Shanghai, China).

### Quantitative Reverse Transcriptase Polymerase Chain Reaction (qRT-PCR)

Total RNA was extracted from 1 × 10^5 ^cells using the RNeasy RNA Mini Kit (Qiagen). First strand cDNA was synthesized using powerscipt reverse transcriptase (Clontech). The following gene-specific primer pairs were used for quantitative PCR:

*ΔNp63*: Forward, 5'-GGGTGAGCGTGTTATTGATGCT-3';

Reverse, 5'-GAGTGGAATGACTTCAACTTT-3'.

*GAPDH*: Forward, 5'-GCTGAGTATGTCGTGGAGTC-3';

Reverse, 5'-AGTTGGTGGTGCAGGATGC-3'.

PCR was performed using a Fast Start Master SYBR Green Kit (Roche) on a LightCycler (Roche). The expression level of *ΔNp63 *mRNA was analyzed using RealQuant software (Roche) and normalized to that of *GAPDH *mRNA.

### Western blot

Cellular proteins were prepared using cell lysis buffer (50 mM Tris-HCl, pH 8.0, 1% NP-40, 2 mM EDTA, 10 mM NaCl, 2 mg/ml aprotinin, 5 mg/ml leupeptin, 2 mg/ml pepstatin, 1 mM DTT, 0.1% SDS and 1 mM phenylmethylsulfonyl fluoride). Equal amounts of protein (50 μg) were separated by 10% SDS PAGE and then transferred to nitrocellulose membranes (NY, USA) by electroblotting. The membranes were blocked with 5% BSA in TBST (10 mM Tris-HCl, pH 8.0, 150 mM NaCl, and 0.05% Tween 20) for 1 hr, and then incubated with mouse anti-human ΔNp63 antibody (Santa Cruz) overnight at 4°C before subsequent incubation with horseradish peroxidase-conjugated goat anti-mouse antibody (BD) for 1 hr at 37°C. Protein was visualized using enhanced chemiluminescence reagent (Santa Cruz). The expression level of ΔNp63 protein was analyzed using LabWork 4.0 program (UVP) and normalized to that of β-actin protein.

### Clonogenic assay

Single-cell suspension was prepared using trypsin treatment. Cells were then seeded into 6-well cell culture plates (200 cells/well) and incubated for 2 weeks at 37°C. Then, cells were washed twice with PBS and stained with a mixture of 6.0% glutaraldehyde and 0.5% crystal violet for 1 hour at 37°C. The plates were air-dried at room temperature. Colony forming efficiency was calculated as the percentage of plated cells that formed colonies.

### Cell population doubling time

Cells were plated into 6-well plates (1 × 10^4 ^cells/well) and cultured at 37°C. Cell population doubling time (PDT) was calculated using the following equation: PDT (hr) = (log2 × t)/(logN_t _- logN_0_), where t = time in culture (hr), N_t _= final cell count, N_0 _= original cell count.

### Cell cycle assay

Cells were fixed in 70% ethanol for 2 hr at 4°C. After washing with PBS, cells were treated with RNaseA (50 μg/ml) and stained with propidium iodide (25 μg/ml) for 30 min at 37°C. Samples were analyzed using an FACSCalibur flow cytometer (BD Biosciences) and distribution of cell-cycle phases was determined using Modfit Software (BD Biosciences). The proliferative index was calculated as the percentage of cells in S/G2/M-phase.

### Apoptosis assay

Cells were stained with annexin V-FITC and propidium iodide using the ANNEXIN V-FITC Kit (Beckman) according to the manufacturer's protocol and subjected to flow cytometric analysis. Viable cells were unstained by annexin V or propidium iodide, early apoptotic cells were stained by annexin V but not propidium iodide, and late apoptotic cells were stained by annexin V and propidium iodide. The apoptotic index was calculated as the percentage of annexin V^+^/propidium iodide^- ^cells.

### Luciferase reporter assay

The full-length 3'-UTR of *ΔNp63 *mRNA containing the miR-203 binding site was amplified by PCR (Forward: 5'-ggggagctcatataagaactcttgcagtct-3'; Reverse: 5'-gggaagcttggtgtacattcttctagaac-3'). Mutant *ΔNp63 *3'-UTR, which carried a substitution of four nucleotides (CTTT to GAAA) within the core binding sites of *ΔNp63 *3'-UTR (14), was obtained using overlapping extension PCR. Normal (or mutant) *ΔNp63 *3'-UTR was cloned into the SacI-HindIII site of the pMIR-REPORT luciferase vector (Biosystems) and named as Luc-ΔNp63 (or Luc-ΔNp63-mut). Then, 1 × 10^6 ^cells were cotransfected with 50 pmol of microRNA-203 (or control microRNA), 1 μg of Luc-ΔNp63 (or Luc-ΔNp63-mut) plasmid, and 1 μg of pMIR-REPORT β-Gal vector using Lipofectamine 2000. The Luciferase activity was examined at 48 hr posttransfection using the luciferase assay kit (Clontech) and normalized to β-galactosidase activity.

### Statistical Analyses

Data are presented as mean ± SEM. Statistical significance was tested using SPSS11.0 software, using *t *tests for 2-group comparisons. A *P *value less than 0.05 is considered statistically significant.

## Results

### MiR-203 could inhibit the proliferation of ESCC cell lines

**Figure 1:**
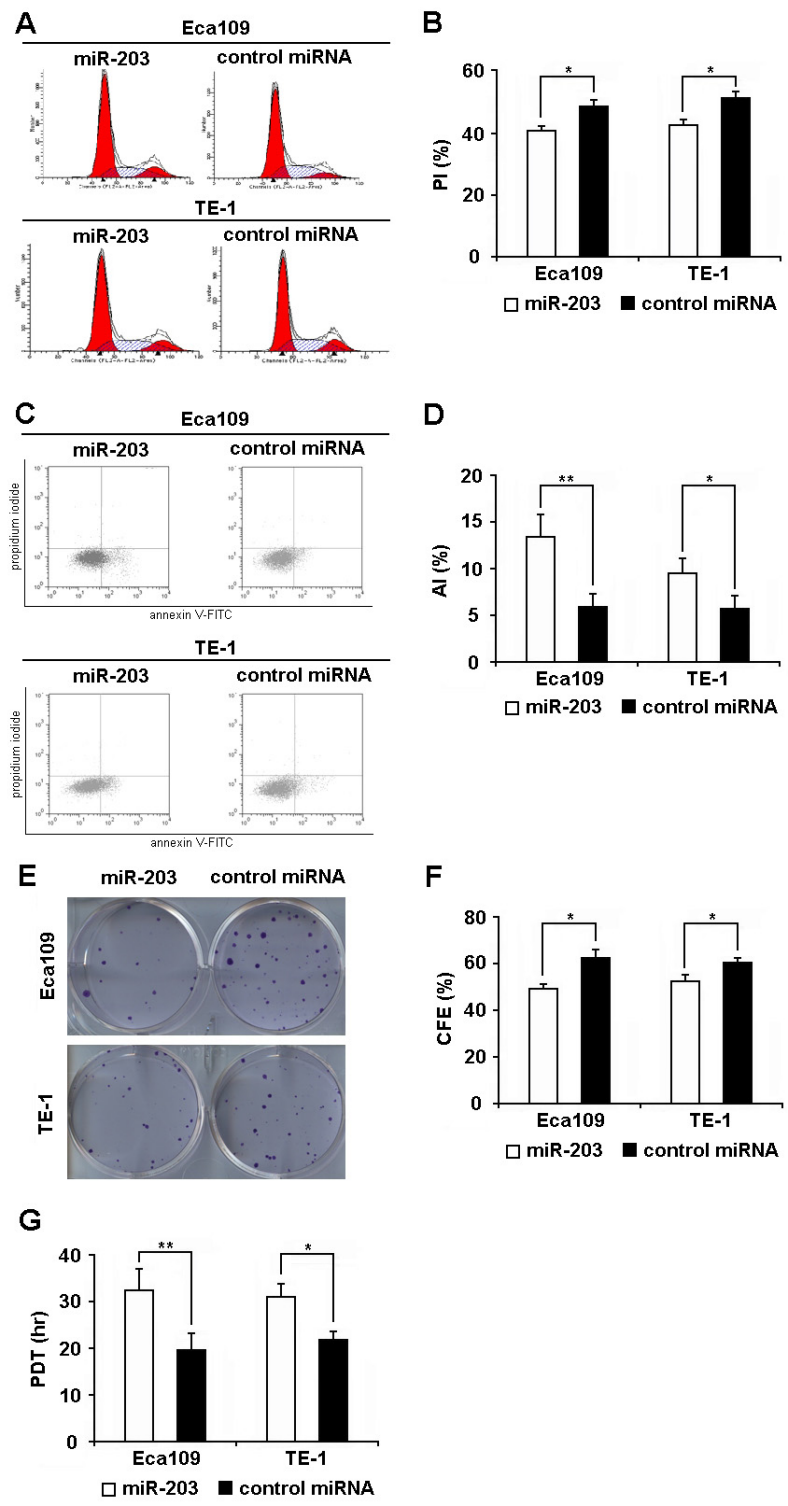
**MiR-203 inhibits the proliferation of ESCC cell lines**. **(A, B) **Cell cycle analysis was performed at 48 hr posttransfection by staining DNA with propidium iodide prior to flow cytometry. Results showed that proliferative index (PI) of cells transfected with miR-203 was significantly lower than that of cells transfected with control microRNA. **(C, D) **Apoptosis assay was performed at 48 hr posttransfection by staining cells with annexin V-FITC and propidium iodide prior to flow cytometry. Results showed that apoptotic index (AI) of cells transfected with miR-203 was significantly higher than that of cells transfected with control microRNA. **(E, F) **Clonogenic assay was performed as described in materials and methods with cells at 24 hr posttransfection. Results showed that the colony forming efficiency (CFE) of cells transfected with miR-203 was significantly lower than that of cells transfected with control microRNA. **(G) **Cell population doubling time (PDT) was determined as described in materials and methods with cells at 24 hr posttransfection. Results showed that the PDT of cells transfected with miR-203 was significantly longer than that of cells transfected with control microRNA. Transfection condition: Cells (1 × 10^6^) of Eca109 and TE-1 were transfected with 50 pmol of miR-203 (or control microRNA). Data represent mean ± SEM from 4 independent experiments; *, P < 0.05 by t test. **, P < 0.01 by t test.

We scanned the basal expression levels of miR-203 in the ESCC cell lines by real time PCR. It was found that both Eca109 and TE-1 expressed very low level of miR-203 (Additional file 1, Figure S1). Therefore, we investigated the effect of miR-203 on cell proliferation using cells transfected with miR-203 (or control microRNA). As shown in Figure S1, mature miR-203 was highly expressed in cells transfected with miR-203 while the expression level of miR-203 was still very low in cells transfected with control microRNA 48 hr posttransfection. In addition, cells treated with miR-203 had a significantly lower proliferative index and a significantly higher apoptotic index than cells transfected with control microRNA (Figure 1A-C and Additional file 1, Table. S1). Meanwhile, cells transfected with miR-203 exhibited significantly lower colony forming efficiency as well as significantly longer population doubling time than those transfected with control microRNA (Figure 1D-F). These results suggest that miR-203 could inhibit the proliferation of ESCC cell.

### Repressing ΔNp63 expression could inhibit the proliferation of ESCC cell

**Figure 2:**
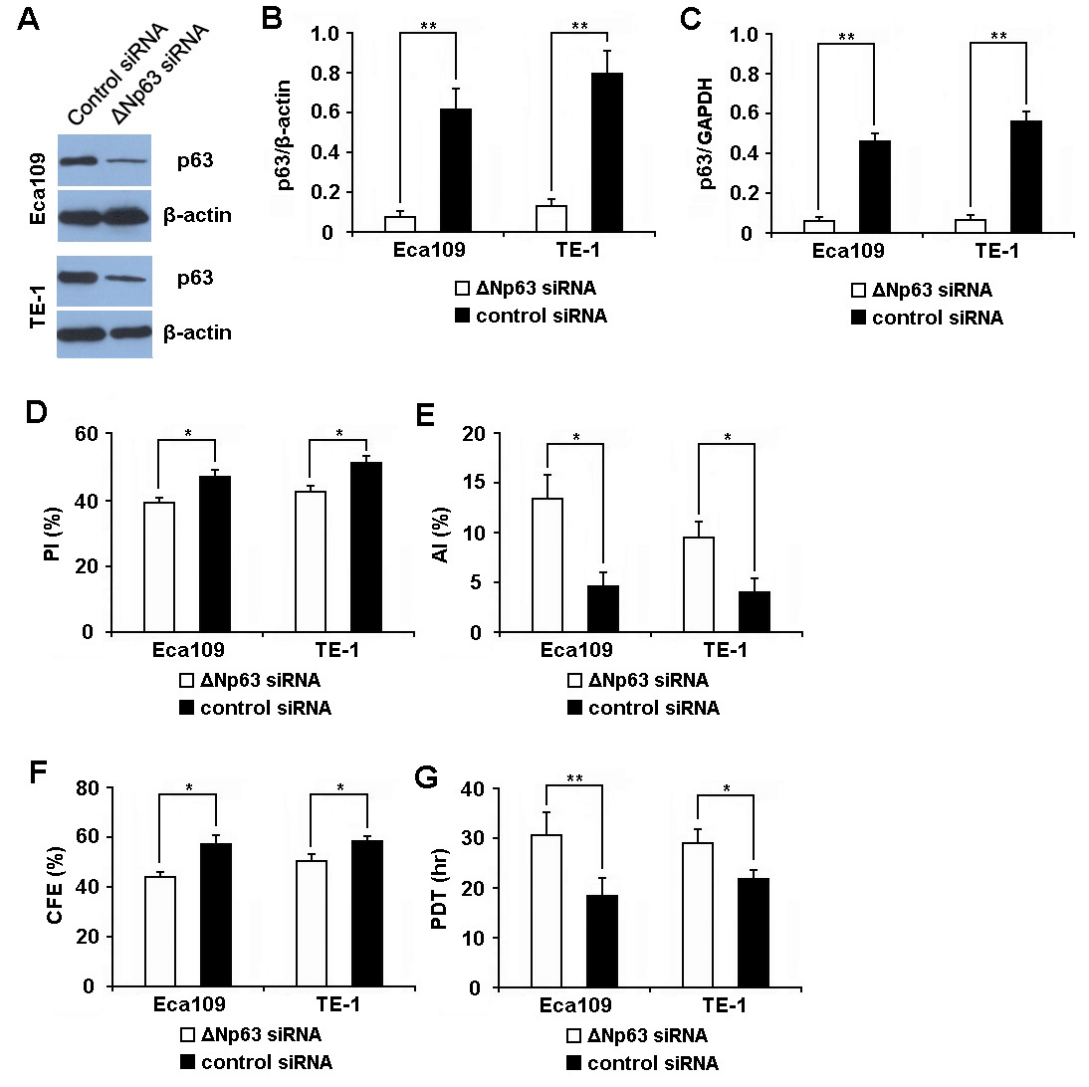
**Repressing ΔNp63 expression inhibits proliferation of ESCC cell lines**. **(A, B) **The expression level of ΔNp63 protein was detected by Western Blot at 48 hr posttransfection and normalized to that of β-actin. Results showed that the expression of ΔNp63 protein was significantly decreased in cells transfected with ΔNp63 siRNA as compared to those transfected with control siRNA. **(C) **The expression level of *ΔNp63 *mRNA was detected by qRT-PCR at 48 hr posttransfection and normalized to that of *GAPDH*. Results showed that the expression of *ΔNp63 *mRNA was significantly decreased in cells transfected with ΔNp63 siRNA as compared to those transfected with control siRNA. **(D-G) **Cell proliferation was evaluated by cell cycle analysis **(D)**, annexin V-FITC/propidium iodide double staining **(E)**, population doubling time determination **(F)**, and clonogenic assay **(G) **as described in materials and methods. Results showed that the proliferative capacity of cells transfected with ΔNp63 siRNA was significantly lower than that of cells transfected with control siRNA. Transfection condition: Cells (1 × 10^6^) of Eca109 and TE-1 were transfected with 50 pmol of ΔNp63 siRNA (or control siRNA). Data represent mean ± SEM from 4 independent experiments; *, P < 0.05 by t test. **, P < 0.01 by t test.

To investigate the effect of ΔNp63 on the proliferation of ESCC cell, we evaluated the cell proliferative capacity of cells (Eca109 and TE-1) transfected with ΔNp63 siRNA (or control siRNA). At 48 hr posttransfection, the expression of ΔNp63 protein and mRNA in the cells transfected with ΔNp63 siRNA was significantly decreased in comparison with that of cells transfected with control siRNA (Figure 2A-C), indicating that the expression of ΔNp63 was effectively inhibited by ΔNp63 siRNA. Subsequent studies showed that the proliferative capacity of cells transfected with ΔNp63 siRNA was significantly lower than that of cells treated with control siRNA. As shown in Figure 2d and 2e, cells transfected with ΔNp63 siRNA showed a significantly lower proliferative index as well as a significantly higher apoptotic index than those cells treated with control siRNA. Meanwhile, cells transfected with ΔNp63 siRNA exhibited a significantly lower colony forming efficiency as well as a significantly longer population doubling time than the control cells (Figure 2F and 2G). These results imply that repressing ΔNp63 expression could inhibit the proliferation of ESCC cell.

### MiR-203 posttranscriptionally down-regulates ΔNp63 expression by targeting the 3' untranslated region of ΔNp63

**Figure 3:**
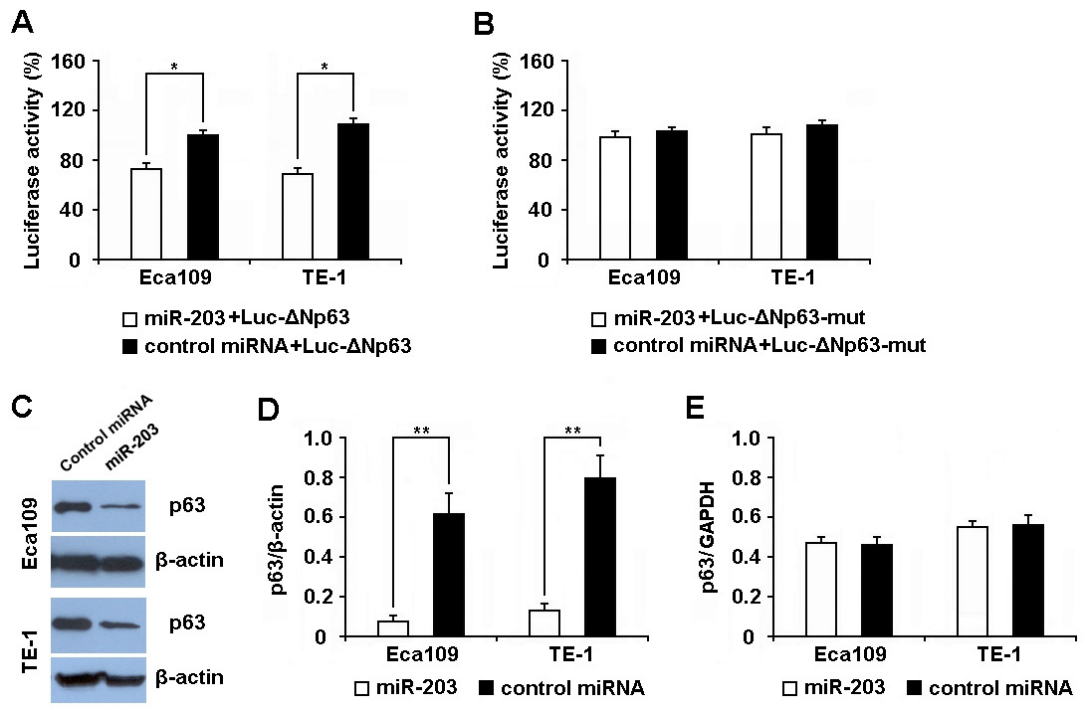
**MiR-203 posttranscriptionally regulates ΔNp63 expression by targeting the 3'-UTR of *ΔNp63***. **(A, B) **1 × 10^6 ^cells (Eca109 or TE-1) were cotransfected with 50 pmol of miR-203 (or control microRNA) and 1 μg of Luc-ΔNp63 (or Luc-ΔNp63-mut) plasmid, respectively. Luciferase reporter assay were performed at 48 hr posttransfection. Results showed that cells cotransfected with miR-203 and Luc-ΔNp63 plasmid exhibited a significant decrease of reporter activity in comparison with those cotransfected with the control microRNA and Luc-ΔNp63 plasmid (a). However, the reporter activity of cells cotransfected with miR-203 and Luc-ΔNp63-mut plasmid showed no significant difference with that of cells cotransfected with control microRNA and Luc-ΔNp63-mut plasmid (b). **(C, D) **Cells (1 × 10^6^) of Eca109 and TE-1 were transfected with 50 pmol of miR-203 (or control microRNA), respectively. The expression level of ΔNp63 protein was detected by Western Blot at 48 hr posttransfection and normalized to that of β-actin. Results showed that the level of ΔNp63 protein was significantly decreased in cells transfected with miR-203 as compared to the cells transfected with control microRNA. Results showed that the expression level of ΔNp63 protein was significantly decreased in cells transfected with miR-203 as compared to the cells transfected with control microRNA. **(E) **Cells (1 × 10^6^) of Eca109 and TE-1 were transfected with 50 pmol of miR-203 (or control microRNA), respectively. The expression level of *ΔNp63 *mRNA was detected by qRT-PCR at 48 hr posttransfection and normalized to that of *GAPDH*. Results showed that the expression level of *ΔNp63 *mRNA exhibited no significantly difference between cells transfected with miR-203 and those transfected with control microRNA. Data represent mean ± SEM from 4 independent experiments; **, P < 0.01 by t test.

It was reported that the 3'-UTR of *ΔNp63 *contains the miR-203 binding site [14]. To determine whether the 3'-UTR of *ΔNp63 *mRNA is a functional target of miR-203 in ESCC cells, we evaluated the reporter activity in cells cotransfected with miR-203 (or control microRNA) and Luc-ΔNp63 plasmid (or Luc-ΔNp63-mut plasmid). As shown in Figure 3A, cells cotransfected with miR-203 and Luc-ΔNp63 plasmid showed a significant decrease of reporter activity in comparison with those cotransfected with the control microRNA and Luc-ΔNp63 plasmid. However, cells cotransfected with miR-203 and Luc-ΔNp63-mut plasmid showed no significant difference in reporter activity as compared with cells cotransfected with control microRNA and Luc-ΔNp63-mut plasmid (Figure 3B).

We further detected the expression of ΔNp63 protein and mRNA by western blot and qRT-PCR in Eca109 and TE-1 cells transfected with miR-203 (or control microRNA). As shown in Figure 3C and 3D, the expression of ΔNp63 protein was decreased by approximately 80% in cells transfected with miR-203 as compared to the cells treated with control microRNA at 48 hr posttransfection (0.08 ± 0.02 vs. 0.62 ± 0.10 for Eca109, P < 0.05; 0.13 ± 0.03 vs. 0.80 ± 0.11 for TE-1, P < 0.05). However, the expression of *ΔNp63 *mRNA showed no significant difference between the 2 groups (Figure 3E). These results indicate that the 3'-UTR of *ΔNp63 *mRNA is a functional target of miR-203 in ESCC cells.

### The inhibition of the proliferation of ESCC cells by miR-203 could be significantly attenuated by the re-expressing of ΔNp63

**Figure 4:**
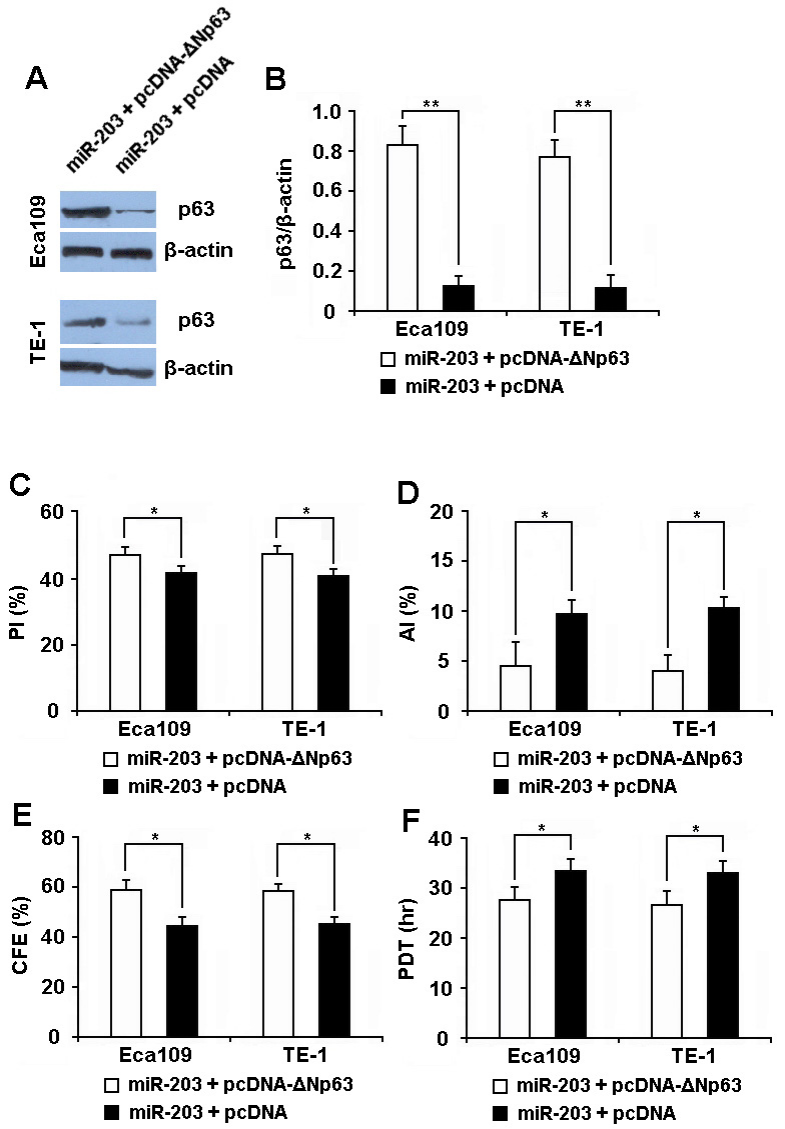
**Re-expressing ΔNp63 could significantly attenuate the effect of miR-203 on the inhibition of ESCC cell proliferation**. **(A, B) **The expression level of ΔNp63 protein was detected by Western Blot at 48 hr posttransfection and normalized to that of β-actin. Results showed that the expression level of ΔNp63 protein was significantly higher in cells cotransfected with miR-203 and pcDNA-ΔNp63 as compared to the cells transfected with miR-203 and empty pcDNA. **(C-F) **Cell proliferation was evaluated by cell cycle analysis **(C)**, annexin V-FITC/propidium iodide double staining **(D)**, population doubling time determination **(E) **and clonogenic assay **(F) **as described in materials and methods. Results showed that the proliferative capacity of cells cotransfected with miR-203 and pcDNA-ΔNp63 was significantly higher than that of cells cotransfected with miR-203 and empty pcDNA. Transfection condition: 1 × 10^6 ^cells (Eca109 or TE-1) were cotransfected with 50 pmol of miR-203 and pcDNA-ΔNp63 (or empty pcDNA). Data represent mean ± SEM from 4 independent experiments; *, P < 0.05 by t test. **, P < 0.01 by t test.

To investigate the functional connection between miR-203 and ΔNp63 in the regulation of ESCC cell proliferation, we further evaluated the proliferative capacity of cells cotransfected with miR-203 and pcDNA-ΔNp63 (or empty pcDNA) plasmid. Notably, the pcDNA-ΔNp63 plasmid was designed to carry the open reading frame of human *ΔNp63 *without 3'-UTR. At 48 hr posttransfection, western blot revealed that the expression level of ΔNp63 in cells cotransfected with miR-203 and pcDNA-ΔNp63 plasmid was significantly higher than that in cells cotransfected with miR-203 and empty pcDNA plasmid (Figure 4A and 4B). Subsequent studies showed that the cells cotransfected with miR-203 and pcDNA-ΔNp63 plasmid had a significantly higher proliferative index, as well as a significantly lower apoptotic index than those cotransfected with miR-203 and empty pcDNA plasmid (Figure 4C and 4D). Meanwhile, cells cotransfected with miR-203 and pcDNA-ΔNp63 plasmid exhibited a significantly higher colony forming efficiency, as well as a significantly shorter population doubling time than the control cells (Figure 4E and 4F). These results imply that re-expressing ΔNp63 could significantly attenuate the inhibitory effect of miR-203 on cell proliferation, suggesting that the miR-203 inhibits the proliferation of ESCC cells through the ΔNp63-mediated signal pathway.

## Discussion

It was known that microRNAs could regulate a variety of cellular pathways by affecting the expression of multiple types of target genes and the alteration of microRNAs expression might contribute to human carcinogenesis [9,11,12]. Thus, an understanding of the specific microRNAs involved in the process of tumor development would provide valuable insight for the diagnosis and treatment of patients with tumor. Here, we have demonstrated that miR-203 could down-regulate the proliferation of ESCC cells, probably through the ΔNp63-mediated signal pathway. Our data suggest that re-expressing miR-203 might benefit the treatment of ESCC.

It was reported that the expression of miR-203 was significantly down-regulated in some tumors, including head and neck squamous cell carcinomas [14], hematopoietic malignancy [13] and colon cancer [15]. Moreover, it was reported that inhibition of miR-203 expression could significantly increase the proliferation of Hela cells [26], whilst re-expression of miR-203 could inhibit the proliferative capacity of cells in human head and neck squamous cell carcinoma [14], hepatocellular carcinoma [16], chronic myelogenous leukemia and B cell leukemia [13]. These findings suggest that miR-203 might function as a tumor suppressor gene in a variety of tumors. In the case of esophageal cancer, genome-wide microRNA expression profile analysis revealed that the expression level of miR-203 was 2- to 10-fold lower in tumor than in the matched normal tissues [19-21]. However, the effect of miR-203 on the cell proliferation in human ESCC has not been reported. In the present study, we found that the proliferative capacity of ESCC cells transfected with miR-203 was significantly lower than that of cells transfected with control microRNA, suggesting that miR-203 could inhibit the proliferative capacity of ESCC cells.

Previous studies indicated that ΔNp63, an alternative splice variant of p63 gene lacking TA domain [17], is the major isotype expressed in a variety of human squamous cell carcinoma including ESCC [27], and that the expression level of ΔNp63 in tumor tissues was significantly higher than in the matched normal tissues [22,27]. In our pilot study, we found ΔNp63 was highly expressed, whilst TAp63 was hardly detectable in Eca109 and TE-1 (Additional file 1, Figure S2). Here, we demonstrated that repressing ΔNp63 expression by siRNA could significantly inhibit the proliferation of ESCC cell lines, implying that ΔNp63 played a positive role in ESCC cell proliferation. Our findings, in combination with the previous reports that ΔNp63 could promote the cell proliferation in head and neck squamous cell carcinoma [14] as well as lung squamous cell carcinoma [27], suggest that ΔNp63 may function as an oncogene in human squamous cell carcinoma.

Recently, using bioinformatic analysis, Lena et al. [14] and Yi et al. [18] independently reported that the 3'-UTR of *ΔNp63 *contain the miR-203 binding site. Subsequent studies showed that miR-203 could repress the expression of ΔNp63 and inhibit cell proliferation in human epithelial precursor cells as well as human head and neck squamous cell carcinoma cells, suggesting that miR-203 is a key molecule controlling the ΔNp63-mediated cell proliferation in some normal and tumor cells [14,18]. However, whether miR-203 regulates the expression of ΔNp63 in ESCC has not been identified before. Here, we showed that miR-203 could significantly inhibit ΔNp63 protein expression without changing the expression level of *ΔNp63 *mRNA, suggesting that miR203 negatively regulated the expression of ΔNp63 at the posttranscriptional level in ESCC. Moreover, we demonstrated that re-expressing ΔNp63 in miR-203 transfected ESCC cells could significantly attenuate miR-203 induced inhibition of cell proliferation. Taken together, our results suggest that miR-203 may function as a tumor suppressor by regulating ΔNp63-mediated signal pathways in human ESCC.

However, we noticed that the proliferative capacity of the ESCC cells cotransfected with miR-203 and pcDNA-ΔNp63 plasmid, though much higher than that of the cells cotransfected with miR-203 and empty pcDNA plasmid, is still significantly lower than that of the cells cotransfected with control microRNA and empty pcDNA plasmid (Additional file 1, Figure S3). This result, combined with the fact that the expression level of ΔNp63 protein in the cells cotransfected with miR-203 and pcDNA-ΔNp63 was significantly higher than that in cells cotransfected with control microRNA and empty pcDNA plasmid (Additional file 1, Figure S4), suggest that miR-203 might regulate the proliferation of ESCC cells through multiple target genes. In this respect, the underlying mechanisms of miR-203 in regulating the proliferation of ESCC cell warrant further investigation.

## Conclusions

In summary, we have demonstrated that miR-203 and ΔNp63 play an important role in ESCC cell proliferation regulation. MiR-203 can significantly inhibit the proliferation of ESCC cell through the ΔNp63-mediated signal pathway. Based on these findings, we propose that miR-203 might be used as a therapeutic agent for ESCC.

## Competing interests

The authors declare that they have no competing interests.

## Authors' contributions

SDH conceived the design of the study and was in charge of its coordination. YY participated in data analysis, performed data interpretation and drafted the manuscript. ZYZ carried out the cell proliferation analysis and helped to draft the manuscript. XHL performed molecular biology experiment and helped to draft the manuscript. DJG participated in cell culture and Luciferase reporter assay. JT participated in flow cytometry analysis. HZC co-conceived the design of the study. All authors read and approved the final manuscript.

## Pre-publication history

The pre-publication history for this paper can be accessed here:

http://www.biomedcentral.com/1471-2407/11/57/prepub

## Supplementary Material

Additional file 1 **Results of the expression of *miR-203*, p63 isoforms (ΔNp63 and TAp63) in Eca109 and TE-1 cell lines; the proliferative capacity of cells cotransfected with miR-203 and pcDNA-ΔNp63 plasmid (or with control microRNA and empty pcDNA plasmid); the expression level of ΔNp63 protein in cells cotransfected with miR-203 and pcDNA-ΔNp63 plasmid (or with control microRNA and empty pcDNA plasmid); cell cycle analysis of Eca109 and TE-1 cell transfected with miR-203 and ΔNp63 siRNA**. Click here for file
