# Supplementary material for: MicroRNA-203 inhibits cell proliferation by repressing ΔNp63 expression in human esophageal squamous cell carcinoma
Source: BMC Cancer. 2011 Feb 7;11:57. doi: 10.1186/1471-2407-11-57 (PMC3044653; doi:10.1186/1471-2407-11-57)
Supplement: Additional file 1 — Results of the expression of miR-203, p63 isoforms (ΔNp63 and TAp63) in Eca109 and TE-1 cell lines; the proliferative capacity of cells cotransfected with miR-203 and pcDNA-ΔNp63 plasmid (or with control microRNA and empty pcDNA plasmid); the expression level of ΔNp63 protein in cells cotransfected with miR-203 and pcDNA-ΔNp63 plasmid (or with control microRNA and empty pcDNA plasmid); cell cycle analysis of Eca109 and TE-1 cell transfected with miR-203 and ΔNp63 siRNA. [file 1471-2407-11-57-S1.DOC]

**Fig. S1 Expression levels of miR-203 in Eca109 and TE-1 cell lines.**

Total RNA was extracted from 1×10^5^ cells using the miRNeasy Mini Kit (QIAGEN). Reverse transcription reactions and real-time PCR reactions were performed using miScript PCR Starter Kit (QIAGEN) and Hs_miR-203_1 miScript Primer Assay system (QIAGEN) according to the manufacturer’s protocol. PCR was performed on a LightCycler and analysis was carried out using RealQuant software. Threshold cycle (*Ct*) values were assigned according to the cycle number at which a fixed fluorescent intensity was achieved. A Δ*Ct* value was calculated by *Ct* (miR-203) - *Ct* (U6). Fold induction of mature miR-203 expression level in the miR-203 (or control microRNA) transfected cells relative to the untreated cells was then calculated as 2^-ΔΔ^*^Ct^*, where ΔΔ*Ct* is the difference between Δ*Ct* (microRNA treated cells) and Δ*Ct* (untreated cells).

**(A-C)** The basal expression levels of miR-203 in Eca109 and TE-1 cell lines were very low.

**(D)** The level of mature miR-203 in Eca109 cells transfected with miR-203 duplex was about 850-fold higher than that in cells transfected with control microRNA or untreated cell.

**(E)** The level of mature miR-203 in TE-1 cells transfected with miR-203 duplex was about 120-fold higher than that in cells transfected with control microRNA or untreated cell.


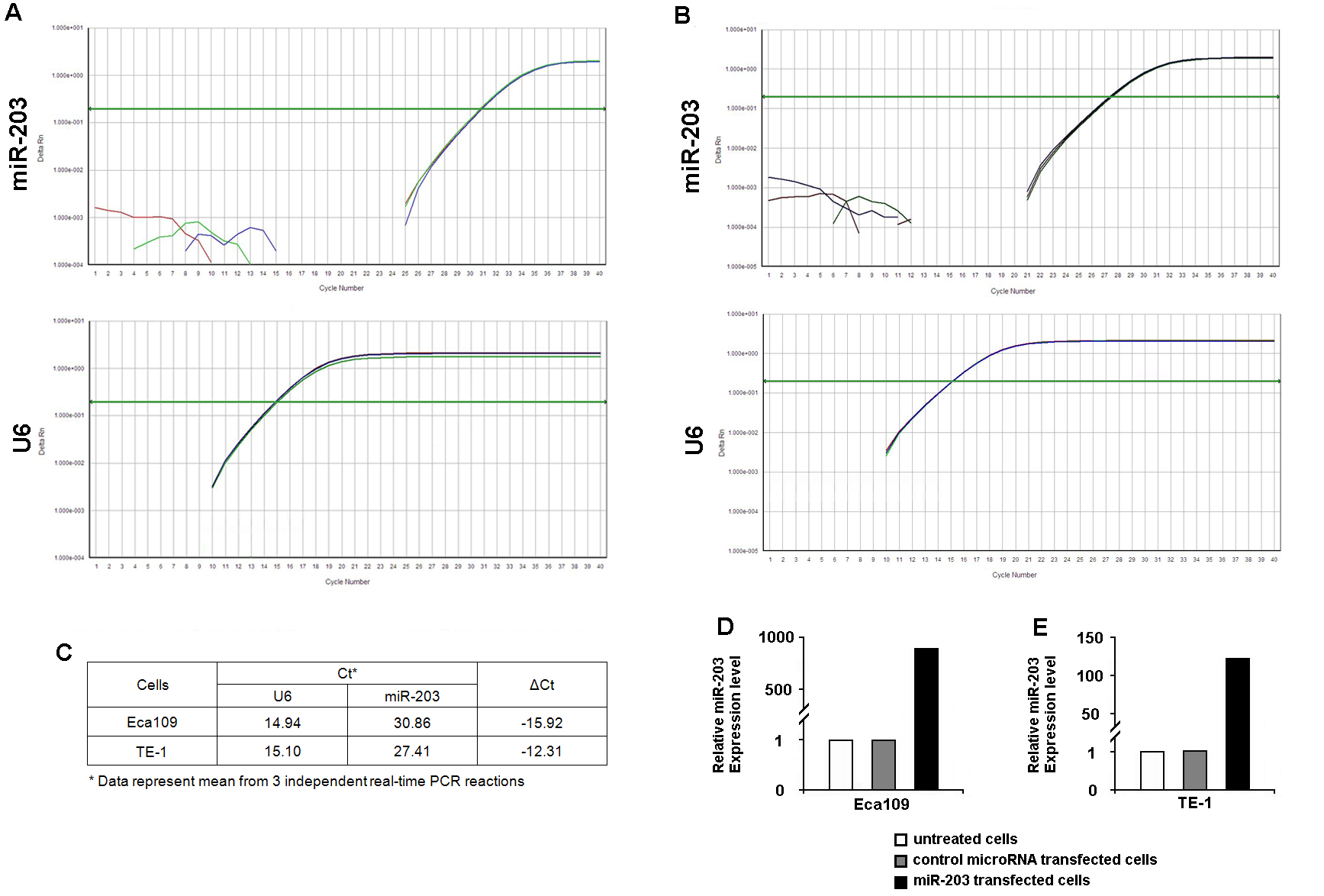


**Fig.S2 The expression level of p63 isoforms (ΔNp63 and TAp63) in Eca109 and TE-1 cell lines.**

Total cell protein was extracted from 1×10^5^ cells, and then western blot was performed as described in Materials and methods. The molecular weight of ΔNp63 and TAp63 were about 72 KDa and 85 KDa, respectively. Results showed that ΔNp63 protein was highly expressed while TAp63 protein was rare in Eca109 and TE-1 cells.


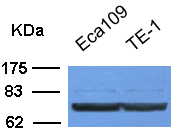


**Fig. S3 The proliferative capacity of cells cotransfected with miR-203 and pcDNA-ΔNp63 plasmid (or with control microRNA and empty pcDNA plasmid).**

**(A-D)** 1×10^6^ cells (Eca109 or TE-1) were cotransfected with 50 pmol of miR-203 and 1 μg of pcDNA-ΔNp63 plasmid (or with 50 pmol of control microRNA and 1 μg of empty pcDNA plasmid). Cell proliferation was evaluated by cell cycle analysis **(A)**, annexin V-FITC/propidium iodide double staining **(B)**, clonogenic assay **(C)** and population doubling time determination **(D)** as described in materials and methods. Results showed that cells cotransfected with miR-203 and pcDNA-ΔNp63 plasmid exhibited significantly lower PI and CFE, whilst significantly longer PDT than those cotransfected with control microRNA and empty pcDNA plasmid in both Eca109 and TE-1.

Data represent mean ± SEM from 4 independent experiments; *, P < 0.05 by t test.


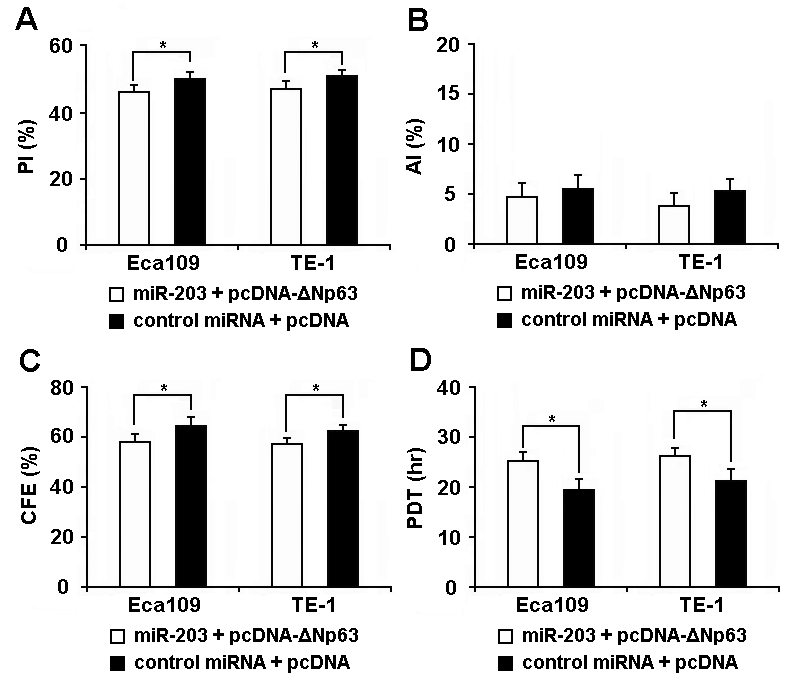


**Fig. S4 The expression level of ΔNp63 protein in cells cotransfected with miR-203 and pcDNA-ΔNp63 plasmid (or with control microRNA and empty pcDNA plasmid).**

**(A, B)** 1×10^6^ cells (Eca109 or TE-1) were cotransfected with 50 pmol of miR-203 and pcDNA-ΔNp63 plasmid (or with control microRNA and empty pcDNA plasmid). The expression level of ΔNp63 protein was detected by Western Blot at 48 hr posttransfection and normalized to that of β-actin. Results showed that the expression level of ΔNp63 protein was significantly higher in cells cotransfected with miR-203 and pcDNA-ΔNp63 plasmid as compared to the cells cotransfected with control microRNA and empty pcDNA plasmid.

Data represent mean ± SEM from 4 independent experiments; *, P < 0.05 by t test.


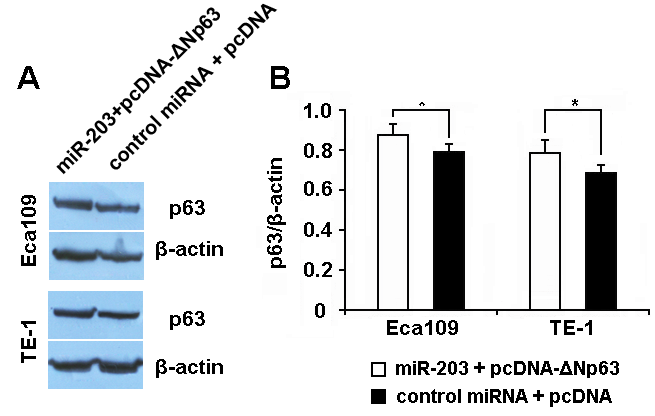


**Table. S1 Cell cycle analysis of Eca109 and TE-1 cell transfected with miR-203 and ΔNp63 siRNA.**

| Cell | Cell cycle | | miR-203 | | Control  microRNA | | *P* | | ΔNp63 siRNA | | Control  siRNA | *P* |
| --- | --- | --- | --- | --- | --- | --- | --- | --- | --- | --- | --- | --- |
|  | G0/G1 | 59.68±1.47 | | 51.53±2.40 | | 0.028 | | 61.13±1.35 | | 53.08±1.97 | | 0.015 |
| Eca109 | S phase | 28.48±1.11 | | 33.38±1.46 | | 0.038 | | 27.13±0.93 | | 32.98±1.87 | | 0.031 |
|  | G2/M | 11.85±0.45 | | 15.10±1.02 | | 0.027 | | 11.75±0.54 | | 13.95±0.68 | | 0.045 |
|  |  |  | |  | |  | |  | |  | |  |
|  | G0/G1 | 58.93±1.55 | | 49.78±2.48 | | 0.020 | | 57.83±1.73 | | 49.13±1.91 | | 0.015 |
| TE-1 | S phase | 29.68±0.87 | | 35.43±1.51 | | 0.016 | | 29.38±1.17 | | 35.05±1.03 | | 0.011 |
|  | G2/M | 11.40±0.72 | | 14.80±1.11 | | 0.042 | | 12.80±0.72 | | 15.83±0.90 | | 0.039 |

Data represent mean ± SEM from 4 independent experiments. Notably, miR-203 and ΔNp63 siRNA could decrease the percentage of cell in both S and G2/M phase.
